# The Ethiopian Cognitive Assessment battery in Schizophrenia (ECAS): a validation study

**DOI:** 10.1038/s41537-024-00462-4

**Published:** 2024-04-06

**Authors:** Yohannes Gebreegziabhere, Kassahun Habatmu, Matteo Cella, Atalay Alem

**Affiliations:** 1 Department of Psychiatry, College of Health Sciences, Addis Ababa University, Addis Ababa, Ethiopia; 2 Department of Nursing, College of Health Sciences, Debre Berhan University, Debre Berhan, Ethiopia; 3 School of Psychology, College of Education and Behavioral Studies, Addis Ababa University, Addis Ababa, Ethiopia; 4 Department of Psychology, Institute of Psychiatry, Psychology and Neuroscience, King’s College London, London, England UK

**Keywords:** Schizophrenia, Psychosis

## Abstract

Cognitive impairment is common in people with schizophrenia (PWS). To detect the presence and its consequences, cognitive measures with sound psychometric properties are needed. However, these are lacking especially in low-income countries. Hence, we developed the Ethiopian Cognitive Assessment battery in Schizophrenia (ECAS). In this study, we evaluated the psychometric properties of the ECAS in a cross-sectional study involving 350 PWS. Confirmatory factor analysis demonstrated a one-factor solution. ECAS score correlated significantly but weakly with a disability measure (*r* = −0.13*, p* = 0.02) and symptom dimensions of PANSS (*r* between −0.12 and −0.29, *p* < 0.05), except for positive symptoms (*r* = −0.10, *p* > 0.05). Years of education (*β* = 0.12, 95% CI (0.09, 0.14), *p* < 0.001), male sex (*β* = 0.22, 95% CI (0.05, 0.39)), age *β* = −0.02, 95% CI (−0.03, −0.01), and medication side effects (*β* = −0.03, 95% CI (−0.06, −0.01), *p* = 0.021) were significantly associated with the composite score of ECAS. The Item Response Theory analysis showed that the tool best functions among participants with moderate cognitive impairment (difficulty coefficient between −1.12 and 0.27). The Differential Item Functioning analyses showed that education had a positive contribution on Digit Symbol Substitution Test (MH OR = 2.64, 95% CI (1.34, 5.20)). The results showed that ECAS is valid in assessing cognition in PWS in low-resource settings.

## Introduction

Cognitive impairment is common in people with schizophrenia (PWS)^1^ which leads to significant burden^2–5^. This calls for routine assessment of cognition. However, cognitive assessment using conventional neurocognitive assessment methods can be challenging, particularly in low-resource settings. This is because the available tests require a longer administration time and special training for test administrators. Recently, shorter measures that can be administered by a wide variety of clinicians with little training were developed and validated in PWS^6,7^, suggesting bright future in the assessment and detection of cognitive impairment in PWS.

Worldwide, several performance-based measures have been validated in PWS. Performance-based measures are those measures in which the examiner provides tasks and scores based on participants’ performance of the tasks. Some examples of validated performance-based measures in PWS include Brief assessment of Cognition in Schizophrenia (BACS)^6^, MATRICS Consensus Cognitive Battery (MCCB)^8^, and Reputable battery for assessment of neuropsychological status^9^. However, adaptation and validation of such measures are lacking in PWS from low- and middle-income countries^10^.

Using cognitive tests developed in the West without proper adaptation and validation in non-Western settings is challenging due to low level of literacy, lack of familiarity with tasks, cultural aspects of sense of time, and contextual differences including availability of norms^11^. Hence, we developed the Ethiopian Cognitive Assessment battery in Schizophrenia (ECAS) following rigorous procedures involving PWS and experts^12^. We followed a four-step instrument selection procedure to select measures^13^. Since we did not find a suitable battery to be adapted as an entity, we selected a test(s) for each domain we agreed to include from different sources to create the ECAS. We used the World List Learning Test (WLLT) to assess verbal memory^14^, Digit Sequencing Task (DST)^6^ and Corsi Block Taping Test (CBTT)^15^ to assess working memory and Animal Naming Test (ANT)^14^ to assess verbal fluency. The remaining three tests, i.e., Digit Symbol Substitution Test (DSST)^16^, Trail Making Tests Part A (TMT A), and Part B (TMT B)^17^, were used to assess attention and speed of processing, and executive function, respectively. The ECAS tests are adapted and modified to be culturally suitable^12^.

The Measurement and Treatment Research to Improve Cognition in Schizophrenia (MATRICS) initiative recommends six criteria for a cognitive battery to be considered for clinical trials^18^. These are comprehensive assessment of cognitive domains, high test-retest reliability, low practice effect, correlation with functional outcome measures, and tolerability and practicality. ECAS addresses four of the seven domains recommended by the MATRICS initiative; it takes about 30 min to administer, has high test-retest reliability, and is tolerable and practical^12^. However, its relationship with functional and symptom measures has not yet been evaluated. In addition, the factor structure of the ECAS was not examined using confirmatory factor analysis (CFA). Therefore, this study aimed to evaluate the validity of ECAS in PWS in Ethiopia.

The overall objective of this study was to investigate the psychometric properties of ECAS and to confirm the findings from the pilot phase in an independent sample. The study also had the following four specific objectives: (1) to confirm the structural validity of the ECAS from the pilot phase using CFA (2) to determine the convergent validity of the measure against a hypothesis in relation to symptom measures, functional measures and selected demographic and clinical variables, and (3) to check the preliminary findings of the item response theory (IRT)-based analysis.

## Methods

### Study design and population

A cross-sectional study was conducted from 23^rd^ January to 27^th^ May 2023. Participants were recruited from a cohort of NeuroGAP-Psychosis Study, a study on Neuropsychiatric Genetics of African Populations with Psychosis conducted preceding our study at Amanueal Mental Specialized Hospital (AMSH) in Addis Ababa, Ethiopia^19^.

PWS participating in the NeuroGAP-Psychosis study aged between 18 and 65 years, who can communicate in Amharic and identify letters and numbers, were included in the study through a consecutive sampling technique. We excluded participants with comorbid diagnoses of a substance use disorder, neurological disorders, organic brain disease, and recent history of head injury with loss of consciousness. Sample size calculation in psychometric studies has no gold standard recommendation^20^. As a rule of thumb, it is recommended to use 10 participants per item of the scale for item-level analysis^21,22^. A sample of greater than 200 participants is recommended for CFA^23^. In support of this, a systematic review of 194 CFA studies reported a median sample size of 389^24^. Considering these recommendations, we decided that 350 PWS would be sufficient for the analysis planned. ECAS has seven tests, and this makes 50 participants per test or item in this case.

### Measures

Data were collected by trained Master of Science (MSc) degree-level mental health clinical officers who have over five years of clinical experience working at AMSH. Data were collected using the following instruments.

#### Sociodemographic and clinical characteristics

We used structured questionnaire to collect sociodemographic data. Using the self-reported structured questionnaire, we collected data about the age of onset, duration of illness (DOI), duration of treatment, and name and frequency of the medications taken. We used a ten-item questionnaire adapted from the Life Chart Schedule (LCS)^25,26^ to collect information about the course of illness and treatment received in the past two years. We also collected data about common antipsychotic side effects using a 17-item questionnaire adapted from a previous study in Ethiopia^27,28^. The LCS and the antipsychotic side effects measure were not validated in Ethiopia. However, they were used in several previous studies and were feasible in the setting^27,28^.

We converted the dosage of different antipsychotics to Chlorpromazine-equivalent defined daily dose (DDD). We used Woods’s^29^ recommendation for atypical antipsychotics, ref. ^30^ recommendation for Clozapine, and the recommendation of Devis for typical antipsychotics^31^. For long-acting antipsychotics, first, we converted them to daily oral doses using ref. ^32^ recommendation, and then we used the above recommendations. Finally, we calculated the total Chlorpromazine-equivalent DDD by summing up the doses of each antipsychotic.

#### Presence and severity of symptoms

We used the Positive and Negative Syndrome Scale (PANSS) to measure the presence and severity of symptoms. The PANSS has 30 items each to be rated on a scale of 1 to 7, with 1 for “Absent” and 7 for “Extreme”^33^. The PANSS is designed to be administered in 30–45 min, with very good to excellent internal consistency coefficient (Cronbach’s alpha of 0.73, 0.83, 0.79 for positive, negative, and disorganized symptom dimensions, respectively)^34^. We used the five-factor solution of PANSS as described in ref. ^35^.

#### Functional status

We used the World Health Organization Disability Assessment Schedule 2.0 (WHODAS-2.0) to measure functional impairment. The WHODAS-2.0 is a cross-cultural measure developed to evaluate the difficulty of a person’s daily activities and social participation^36^. It is translated into 47 languages and dialects and used in 27 health conditions, of which 40% are mental health conditions^37^. Both the 12 and 36-item versions of WHODAS-2.0 have been adapted and validated in Ethiopia. The Amharic version of WHODAS-2.0 was found to have good psychometric properties including excellent internal consistency reliability (Cronbach’s alpha between 0.88 and 0.98 for sub-scales)^38^. In this study, we used the 12-item version of WHODAS-2.0 and summed the items to create a total score.

#### Cognitive status

We used ECAS to assess participants’ cognitive status^12^. ECAS assesses six domains: verbal memory with WLLT, working memory with DST and CBTT, and verbal fluency with ANT. DSST, TMT A, and TMT B are designed to assess attention and speed of processing, and executive function, respectively. DST is from BACS and is the property of WCG. The tasks in each test are described in detail in supplementary material 1. We standardized each test score using the mean and standard deviation (SD) we obtained from controls in the previous phase of the study^12^. The ECAS is found to have good test-retest reliability and internal consistency reliability (Cronbach’s alpha = 0.81)^12^.

### Data analysis

We coded and double-entered the data into EpiData version 4.6.0.6 software. The data were then exported into Stata version 17 and R statistical software for analysis. The details of the methods of analysis we used are presented below.

#### Structural validity

Before deciding to use CFA, the tool is required to be reflective. We assumed that the latent variable (i.e., cognition) caused the measured variables (the tests in the battery). Then, we fitted CFA to examine the structural validity of ECAS following CFA guidelines^39,40^.

First, we decided on the number of factors and variables that load to each factor based on a previous exploratory factor analysis (EFA) conducted in a similar population^12^ and specified the measurement model. The previous EFA analysis and our theoretical assumption suggest one latent variable of cognition with no correlations between error terms. We have confirmed that the number of the variance-covariance matrix is greater than the number of parameters estimated (i.e., the model is over-identified).

Then, we fitted CFA by fixing the scale of the latent variable; to do so, we fixed the factor loading of the first test to 1. Finally, we estimated CFA using the diagonal weighted least square estimation method since the tests in ECAS did not fulfill the multivariate normality assumption of the maximum likelihood estimation method^41,42^. We used Mardia’s test to check multivariate normality^43^. We used the Lavaan package of R statistical software to conduct this analysis.

After the estimation, we tested whether the model fitted the sample covariance matrix. First, we confirmed that the model converges, and the parameters estimated are within the acceptable range (i.e., variables with the same expression have the same sign of factor loading, factor loadings are between -1 and 1, and no negative coefficient for error terms). Since both conditions were satisfied, we tested the model using model fit indices. We used Hu and Bentler’s^44^ recommended cut-offs to decide on fitness of the model: a non-significant chi-square test from the absolute fit index, Comparative Fit Index (CFI) close to 0.95 or higher, Root Mean Square Error of Approximation close to 0.06 or lower, and Standardized Root Mean square Residual close to 0.08 or lower, and Tucker-Lewis’s index (TLI) values close to 0.95 or higher.

#### Hypothesis testing

We determined the convergent validity of ECAS by correlating its scores with the scores of tools that measure constructs that theoretically are assumed to correlate with cognition (functionality and symptom dimensions). Since all the variables are continuous, assumed to have a nearly linear relationship, and have no significant outliers, we used the Pearson correlation coefficient (*r*)^45,46^.

In addition, we determined the association between the composite score of ECAS and factors commonly reported to be associated with cognitive impairment in PWS. We used a hypothesis-driven approach to conduct multiple linear regression. In the final model, we included sex, age, and years of education from demographic variables and DOI, chlorpromazine equivalent DDD, and the number of medication side effects from clinical variables. We checked the assumptions for multiple regression i.e., linearity of the relationship between the dependant and independent variables, homoscedasticity, collinearity/multicollinearity, and normality of residuals^39,47^. We used standardized coefficients to compare the strength of association across the variables in the model. We assessed the overall model fitness in predicting the dependent variable using a significant *F*-test and a higher adjusted coefficient of determination (*R*^2^).

#### Item response theory (IRT) based analysis

To determine the difficulty and discrimination indices of the ECAS, we conducted an item response theory (IRT)-based analysis^22,48^. We checked the assumptions of IRT, i.e., unidimensionality, local independence, and monotonicity^49^.

We decided to use a unidimensional two-parameter logistic (2pl) IRT model as this is appropriate for the current study, considering the dimensionality, objective of the study, sample size, and response category^49^. The tests in the battery produce continuous outcomes; however, there is no IRT model for continuous variables. Therefore, we categorized each test based on the cut-off scores from the receiver operating characteristic curve analysis conducted in the previous study^12^. After categorization, we fitted a 2pl IRT model, where two of the three parameters (i.e., difficulty and discrimination) were estimated^48–50^.

Finally, using a loglikelihood ratio test and Akaike’s information criterion (AIC), we checked if the chosen model (i.e., 2pl IRT model) fits the data better than a more restrictive model (i.e., one-parameter logistic (1pl) IRT model). The null hypothesis for loglikelihood test was that the restrictive model (i.e., 1pl) best fits the data and the lower the AIC, the better fits the data.

#### Differential Item Functioning (DIF)

Item bias or differential item functioning (DIF) is the unfairness of the items/tests towards sub-groups of participants^51^. Since we suspected that participants with the same ability might perform differently because of certain variables, we conducted a DIF analysis concerning educational status. We conducted both uniform and non-uniform DIF. For tests that showed uniform DIF, it is possible to quantify the amount and direction of bias. As a result, we conducted the Mantel—Haenszel (MH) DIF analysis for those that showed uniform DIF. Since DIF needed to be conducted in categorical variables, we used the cut-off from the ROC-curve analysis from the previous study to categorize each test into two^12^. For the educational characteristics, we categorized the participants’ educational status into two groups: ≥11 (a reference group) and less than 11 years of education.

## Results

### Characteristics of participants

Three-hundred fifty PWS (27.7% female) were involved in this study. For details of the characteristics of participants, see Table 1. In the last two years, 45.1% (*n* = 158) of the participants were in remission, with most reporting complete remission (70.89%, *n* = 112). Over three-fourths (76.0%, *n* = 266) of the participants reported more than one medication side effect, with a mean of three side effects ranging from 0 to 12. The mean time taken to administer and score the ECAS tests was 33.1 ± 8.07 min, ranging from 2.2 min for ANT to 13.3 min for DSST.

**Table 1:** Socio-demographic and clinical characteristics of participants.

Socio-demographic characteristics		Frequency (*n* = 350)
Sex, % male		72.3
Age in years, mean (SD.)		37.3 (10.1)
Education in years, mean (SD.)		11.1 (3.4)
Marital status, %	Single	65.4
	Married	19.1
	Separated	7.1
	Other^c^	15
Occupational status, %	Unable to work	10.6
	Unemployed	29.7
	Private business	29.4
	Government employee	11.4
	Student	4.3
	Housewife	4.0
	Farmer	2.6
	NGO employee	1.7
	Other^d^	6.3
Monthly income in USD^a^, Median (IQR)		64.1 (36.6, 109.9)
Relative wealth, %	Low	60.9
	Medium	31.7
	High	7.4
Religion, %	Orthodox	58.3
	Muslim	23.1
	Protestant	17.1
	Other^e^	1.4
Residence, % urban		89.7
Clinical characteristics		
Age of onset in years, mean (SD.)		25.0 (7.69)
Duration of illness in years, mean (SD.)		12.3 (8.9)
Years on treatment in years, mean (SD.)		10.8 (8.5)
Chlorpromazine equivalent DDD in mg/day, mean (SD.)		387.8 (514.4)
Number of side effects reported, mean (SD.)		3.0 (3.0)
Number of admissions^b^, %	0	71.1
	1	18.9
	2	6.9
	3 or 4	3.1
Types of antipsychotics, %	Single atypical	34.6
	Single typical	20.0
	Both typical and atypical	7.7
	Different combination	37.7
Positive symptoms, mean (SD.)		10.56 (6.08)
Negative symptoms, mean (SD.)		11.98 (5.80)
Disorganized symptoms, mean (SD.)		13.87 (5.11)
Excitement symptoms, mean (SD.)		10.29 (3.91)
Emotional symptoms, mean (SD.)		11.57 (5.23)
WHODAS-2.0 total score, mean (SD.)		20.62 (9.97)

*DDD* Defined Daily Doze, *DOI* Duration of illness, *ETB* Ethiopian Birr, *IQR* Inter Quartier Range, *NGO* None Government Organization, *SD* Standard Deviation, *USD* United States Dollar, *WHODAS-2.0* World Health Organization Disability Assessment Schedule 2.0.

^a^1 USD ≈ 54.6 ETB during the study period.

^b^Assessed for the last two years.

^c^Separated, divorced, and widowed.

^d^Daily laborer and Pension.

^e^Catholic (*n* = 2), Jehovah’s Witness (*n* = 2), Rastafari (*n* = 1).

### Structural validity

**Fig. 1:**
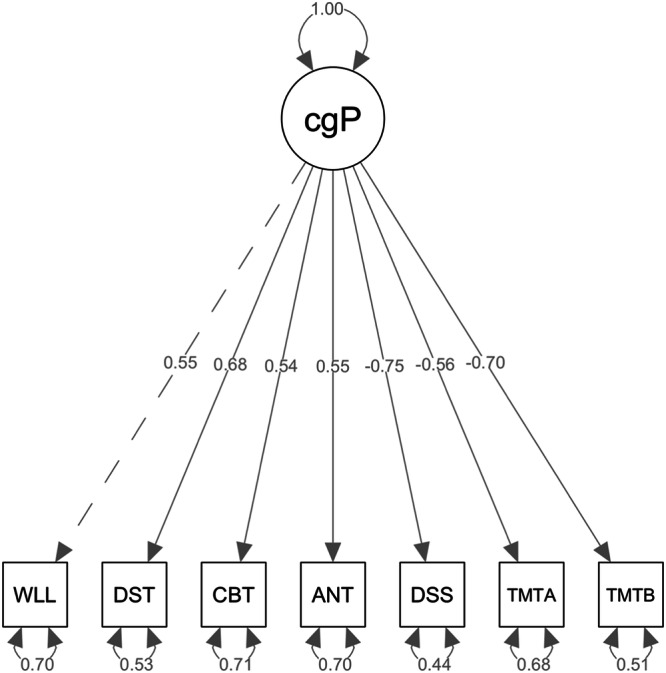
Factor loadings of the one-factor model confirmatory factor analysis of the Ethiopian Cognitive Assessment battery in Schizophrenia (ECAS). This figure showed the one factor structure obtained from a CAF-analysis, with high factor loadings between 0.55 and 0.75. In the figure the following abbreviations are used. ANT Animal Naming Test, CBT Corsi Block Taping test, CgP a latent construct of cognition based on performance-based tests, DSS Digit Symbol Substitution test, DST Digit Sequencing Tests, TMTA Trail Making Test Part A, TMTB Trail Making Test Part B, WLL Word List Learning test.

The CFA revealed that ECAS adequately reflects the unidimensionality of cognition in PWS. The one-factor model presented in Fig. 1 showed that the standardized factor loadings are in the expected direction, and all are significant. We noticed that none of the error terms were negative. All model fit indices suggested excellent fit (Table 2).

**Table 2:** Fit indices and criteria for a good fit of the confirmatory factor analysis of the Ethiopian Cognitive Assessment battery in Schizophrenia (ECAS).

Type of Fit indices	Model fit indices	Criteria for good fit^a^
Chi-square test	0.067	*P* > 0.05
CFI	0.990	Close to 0.95 or higher
TLI	0.984	Close to 0.95 or higher
RMSEA	0.042	Close to 0.06 or lower
SRMR	0.083	Close to 0.08 or lower

*CFI* Comparative fit index, *RMSEA* Root mean square error of approximation, *SRMR* Standardized root mean square residual, *TLI* Tucker-Lewis’s index.

^a^Criteria for good fit are based on Hu and Bentler’s.

### Hypothesis testing

A weak but significant correlation was found between the total score of WHODAS-2.0 and the composite score of ECAS (*r* = −0.13, *p* = 0.02). We found a significant but weak correlation between the composite score of ECAS and the five symptom dimensions of PANSS (*r* ranging from −0.12 to −0.29, *p* < 0.05), except for the positive symptom dimension (*r* = −0.10, *p* > 0.05). A relatively higher correlation was found between the ECAS and the disorganized and negative symptom dimensions (Table 3).

**Table 3:** Pearson correlation of the scores of each test and the composite score of the Ethiopian Cognitive Assessment battery in Schizophrenia (ECAS) with functional disability and symptom severity.

Tests	WHODAS-2.0	PANSS Symptom dimensions				
		Positive	Negative	Disorganized	Excitement	Emotional
WLLT standardized	−0.16**	−0.04	−0.20***	−0.22***	−0.14*	−0.13*
DST standardized	−0.18**	−0.20***	−0.26***	−0.32***	−0.22***	−0.22***
CBTT standardized	−0.12*	−0.13*	−0.16**	−0.27***	−0.15**	−0.12*
ANT standardized	−0.19***	−0.10	−0.21***	−0.20***	−0.11*	−0.13*
DSST standardized	−0.08	−0.12*	−0.19***	−0.26***	−0.18***	−0.12*
TMT: Part A standardized	0.02	0.02	−0.03	−0.07	−0.02	0.04
TMT: Part B standardized	−0.00	−0.00	−0.07	−0.14	−0.07	−0.00
Composite score of ECAS	−0.13*	−0.10	−0.21***	−0.29***	−0.17**	−0.12*

*ECAS* Ethiopian Cognitive Assessment battery in Schizophrenia, *PANSS* Positive and Negative Syndrome Scale, *WHODAS-2.0* World Health Organization Disability Assessment Schedule version 2.0.

*p < 0.05, **p < 0.01, *** for *p* < 0.001.

All the assumptions of multiple regression were fulfilled except homoscedasticity. Since the homoscedasticity assumption was not fulfilled, we conducted the multiple regression analysis with a robust estimation method instead of the default ordinary least square estimation method. As shown in Table 4, sex, age, years of education, and number of medication side effects were significantly associated with the composite score of ECAS. Males scored 0.22 points higher in the composite score of ECAS than females (*β* = 0.22, 95% CI (0.05, 0.39), *p* = 0.010). For every one-year increase in age, the composite score of ECAS decreases by 0.02 points (*β* = −0.02, 95% CI (−0.03, −0.01), *p* = 0.005). As the year of education increases by one, the composite score of ECAS also increases by 0.12 points (*β* = 0.12, 95% CI (0.09, 0.14), *p* < 0.001). When the number of medication side effects reported by the participants increases by one, the composite score of ECAS decreases by 0.03 points (*β* = −0.03, 95% CI (−0.06, −0.01), *p* = 0.021). As the standardized coefficient suggested, the strongest relationship is with the years of education of the participant followed by the age of the participants.

**Table 4:** Association of selected sociodemographic and clinical characteristics with the composite score of the Ethiopian Cognitive Assessment battery in Schizophrenia (ECAS).

Variables	Unstandardized *β* Coefficient (95% Confidence Interval)	*p* value	Standardized *β* Coefficient
Sex	0.22 (0.05, 0.39)	**0.010**	0.12
Age in years	−0.02 (−0.03, −0.01)	**0.005**	−0.22
Years of education	0.12 (0.09, 0.14)	**<0.001**	0.50
Chlorpromazine equivalent DDD	−0.00 (−0.00, 0.00)	0.105	−0.08
Number of medication side effects	−0.03 (−0.06, −0.01)	**0.021**	−0.12
DOI in years	0.01 (−0.01, 0.02)	0.295	0.07

*DDD* daily defend dose, *DOI* Duration of illness

The adjusted coefficient of determination (adjusted *R*^2^) value is 0.30, indicating that the predictor variables in the model explain 30% of the variance in the composite score of ECAS. Also, the *F*-statistics showed that the model is significant (*p* < 0.001), supporting the overall fitness of the model.

### Item Response Theory (IRT) based analysis

We found none of the tests to have a discrimination coefficient above 4, and as the test characteristic curve (TCC) showed, the expected score increased when the ability increased. Therefore, we can confirm that the local independence and monotonicity assumptions were fulfilled.

Table 5 presented the tests in ascending order based on their difficulty; all are significant except DSST. Similarly, the discrimination parameters were significant.

**Table 5:** Item parameters of tests of the Ethiopian Cognitive Assessment battery in Schizophrenia (ECAS) and differential item function analysis sorted based on their difficulty coefficient in descending order.

Name of the test	IRT Parameters		DIF analysis by educational status (*p* value)	
	Difficulty (95% CI)	Discrimination (95% CI)	Nonuniform DIF	Uniform DIF
CBTT	0.27 (0.03, 0.51)*	1.16 (0.77, 1.55)***	**0.006**	0.544
DSST	0.03 (−0.11, 0.18)	3.57 (1.71, 5.42)***	0.752	**0.006**
WLLT	−0.64 (−0.93, −0.36)***	1.11 (0.74, 1.49)***	0.836	0.554
ANT	−0.71 (−1.05, −0.38)***	0.94 (0.59, 1.28)***	0.941	0.315
TMT A	−0.72 (−0.94, −0.50)***	1.81 (1.23, 2.39)***	0.948	0.442
TMT B	−0.90 (−1.11, −0.68)***	2.37 (1.54, 3.19)***	0.527	0.783
DST	−1.12 (−1.39, −0.84)***	1.88 (1.23, 2.53)***	0.558	0.504

*ANT* Animal Naming Test, *CBTT* Corsi Block Taping Tests, *DIF* Differential Item Functioning, *DSST* Digit Symbol Substitution Test, *DST* Digit Sequencing Test, *TMT A* Trail Making Test Part A, *TMT B* Trail Making Test Part B, *WLLT* Word List Learning Test.

* for *p* < 0.05; *** for *p* < 0.001.

Bold is for *p* < 0.05.

**Fig. 2:**
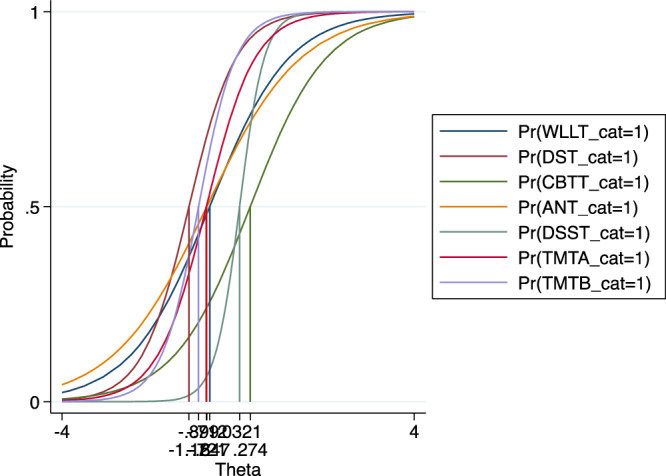
Item Characteristic Curve (ICC) for the tests of the Ethiopian Cognitive Assessment battery in Schizophrenia (ECAS). This figure showed that the difficulty level of each test of ECAS was among participants with moderate impairment. The figure is based on a two-parameters logistic item response analysis.

**Fig. 3:**
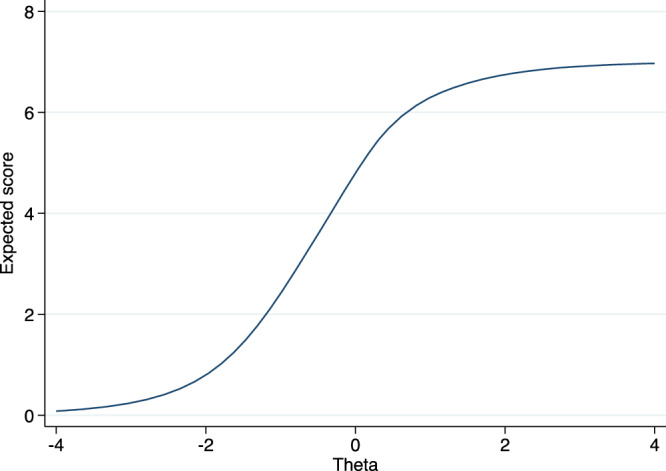
Test Characteristic Curve (TCC) of the Ethiopian Cognitive Assessment battery in Schizophrenia (ECAS). This figure showed that the difficulty level of the battery was among participants with moderate impairment. The figure is based on a two-parameters logistic item response analysis.

The item characteristic curve graph for all the tests is concentrated at the center, suggesting that the tests have moderate difficulty level (Fig. 2). Similarly, the TCC graph is not shifted to the right or left that means the battery as a sum also has a moderate difficulty level (Fig. 3).

**Fig. 4:**
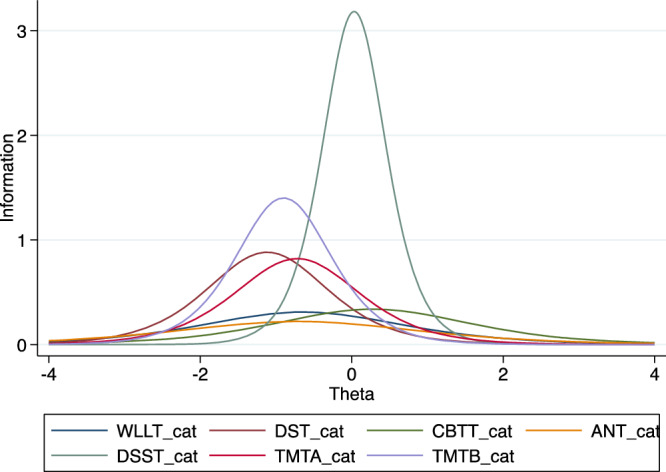
Item Information Function (TIF) for the tests of the Ethiopian Cognitive Assessment battery in Schizophrenia (ECAS). This figure showed that the discrimination parameter for DSST is higher and all of the tests give much information among participants with moderate impairment. The figure is based on a two-parameters logistic item response analysis.

**Fig. 5:**
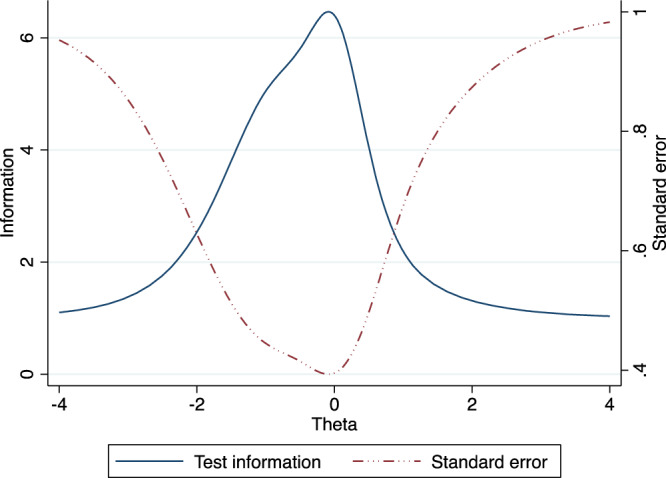
Test Information Function (TIF) and standard error of tests of the Ethiopian Cognitive battery in Schizophrenia (ECAS). This figure showed that the battery works best among participants with moderate impairment. The figure is based on a two-parameters logistic item response analysis.

The item information function (IIF) graph for all the tests is at the center with DSST giving the higher information suggesting that it has a higher discrimination coefficient (Fig. 4). Again, the peak of the test information function (TIF) is at the center, suggesting that the measure best functions among participants with a medium latent ability (moderate cognitive impairment) (Fig. 5).

Finally, we checked if the chosen model (i.e., 2pl IRT model) fits the data better than a more restrictive model (i.e., 1pl IRT model) using the loglikelihood test and AIC. We found that the chi-square test for the loglikelihood difference was significant (*p* < 0.001), and AIC was lower for the 2pl model (i.e., 2733.44 for 1pl vs 2703.80 for 2pl). Therefore, we rejected the null hypothesis and concluded that the 2pl IRT model better fits the data.

### Differential Item Functioning (DIF)

Regarding educational status, none of the tests showed non-uniform and uniform DIF except CBTT and DSST (Table 5). CBTT showed a non-uniform DIF (*p* = 0.006), while DSST showed a uniform DIF (*p* = 0.006). We found that the odds of those with lower educational status needing more time to complete DSST was 2.64 times higher than those with years of education above 11 (MH OR = 2.64, 95% CI (1.34, 5.20), *p* = 0.008).

## Discussion

The findings of this study showed that ECAS is a brief instrument to administer and easy to score, with both these processes taking approximately 30 min, similar to the time taken in the previous study^12^. The DSST took the largest proportion of administration and scoring time (12.3 min). A possible change to reduce administration time for this test is changing the scoring procedure of DSST from time to complete to the number of boxes with correct number-symbol pairs in 120 s.

The CFA analysis confirmed the one-factor structure of ECAS. This demonstrated that the battery measures a dominant factor accounting for the different domains of cognitive impairment. This aligns with previous studies showing that a dominant cognitive factor could account for domains of cognition impaired in PWS^52–55^. This suggests using the composite score of ECAS, as we previously put forward^12^. Having a composite score helps the battery to be less complex in understanding the cognitive impairment in each participant. It can also help clinicians/researchers compare participants’ cognitive status with different domain-level impairments under one umbrella (composite score).

Regarding convergent validity, we found a weak correlation between the scores of each test in the battery and the composite score of ECAS with the scores of WHODAS-2.0. Previous studies reported a similar finding to what we reported in the current study. Performance-based cognitive measures were reported to have a weak to moderate correlation with self-reported functional measures^56–59^. More specifically, previous studies found a weak correlation between performance-based cognitive measures and global assessment of functioning (GAF)^54,60–63^. Another study from North India reported a weak to moderate correlation between WHODAS-2.0 scores and the composite score of BACS^64^. One possible reason for this weak correlation is that functioning is a broad concept and includes involvement in personal, family, and social activities, and this is not captured by the cognitive assessment methods. Secondly, most studies collected data using a cross-sectional study design. Since both conditions fluctuate with time and symptom severity, especially functioning, a follow-up study might provide a better picture of the relationship.

Previous studies showed a weak to moderate correlation between cognitive performance as measured with performance-based tests with negative and disorganized symptoms assessed using PANSS but no correlation with positive symptoms^65–67^. This is similar to our finding that the composite score of ECAS has no association with the positive symptom dimension and a weak correlation with the negative and disorganized symptom dimensions. These findings support the notion that cognitive symptoms and other symptom dimensions have limited overlap, especially with positive symptom dimension. This suggests that due attention should be given to assessing and treating cognitive impairment in PWS.

We found that the composite scores of ECAS are associated with sex, age, years of education, and medication side effects as hypothesized. This is also observed in the DIF analysis, which shows that DSST favors participants with better educational status. Our finding aligns with previous studies that used BACS, MCCB, and other batteries^54,68–70^. This might be because education is related to knowledge acquisition, which might increase sensitization and adaptation to cognitive tests such as reading, listening, communication, and examination processes one way or another. We recommend future studies to develop sex, age, and educational status-specific norms for wider and unbiased use of ECAS. Further improvement of the tests of ECAS to make them less biased in terms of those factors would be another potential research area. A possible change for tests that showed DIF includes changing the scoring of DSST from time to completion to number of correct box-shape pairs in 120 s, and for CBTT increasing the number of steps to be followed.

The IRT-based analysis confirmed what we found in the previous study, which found that the tool best functions among participants with moderate impairment^12^. This finding highlights that IRT-based analyses are not sample dependent, unlike the classical test-based analysis, where the parameters change whenever the sample characteristic changes. In item development it is recommended to include items from different difficulty levels, however, usually it is ideal to find a tool that works across all the difficulty levels. Considering the number of tests and the duration of administration of ECAS, it is less realistic to expect the tool to give information across the difficulty levels. Hence, depending upon the objective of the study, it will be more appropriate to use ECAS to assess cognitive impairment among participants with moderate impairment.

One of the strengths of this study is that we used a large sample of PWS from a low-income setting, which is rare in validation studies of cognitive measures. We also evaluated the correlation of ECAS with functional and symptom measures, which was missing during the development of the ECAS. Furthermore, we used advanced statistical techniques to uncover the objectives of the study. This study is the first to validate a contextually adapted cognitive measure in the African setting, which showed comparable results to non-western settings. However, the following methodological limitations should be considered while interpreting the findings of this study.

Since there is no normative reference, we used a control group’s mean and standard deviation, explicitly designed to be comparable with PWS in the pilot study, to calculate standardized scores. This might limit the interpretation of the findings in this study. Participants in this study had an average of 11 years of education, which might not be representative of PWS in Ethiopia, especially those from rural areas.

Nevertheless, the study has useful implications for clinicians, researchers, and experts in the area. Clinicians can take advantage of the short administration and cultural appropriateness of the ECAS. Although there are no approved pharmacological interventions for cognitive impairment in PWS, so far, depending on individual patient performance, clinicians can use this tool to choose/avoid medications reported to improve/worsen cognitive function. There are proven psychosocial approaches for cognitive difficulties, such as cognitive remediation^71^. Hence, clinicians can use the tool to identify patients needing therapy. Clinicians can also follow their patients using the battery and see if they need a specific treatment plan. This can be used in managing the condition, including family education, where the cognitive impairment needs to be seen as part of the disease. Hence, it helps to reduce stigma and increase support in daily tasks according to the patient’s needs.

The current study can be a steppingstone for cognitive function research in low-income settings. Researchers interested to evaluate changes in cognitive status over time or pre-post studies of different interventions can take advantage of this battery. Nowadays, technological advancement is progressing fast, and the healthcare industry is utilizing innovations such as artificial intelligence-supported assessment and diagnostic methods to improve the access to assessment and therapy. A potential future ambition for this tool could be to develop a digital version of this test for easy, accurate, and faster administration.

There has been a push to include cognitive impairment as one criterion in the diagnosis of Schizophrenia^72,73^. One of the challenges raised against this is the lack of appropriate cognitive measures across settings, especially in resource-scarce settings. With ECAS and hopefully other similar batteries, experts can now consider an evaluation of cognitive symptoms in the diagnostic criteria for schizophrenia. This study demonstrated that a culture and context-appropriate cognitive battery is worthy and leading to convergent findings to studies conducted in high-income countries.

### Supplementary information

Supplementary material 1

## Data Availability

All the data used is made available in the manuscript and supplementary materials. Additional information can be available from the corresponding author upon reasonable request.
