# Supplementary material for: The Ethiopian Cognitive Assessment battery in Schizophrenia (ECAS): a validation study
Source: Schizophrenia (Heidelb). 2024 Apr 6;10(1):42. doi: 10.1038/s41537-024-00462-4 (PMC10998832; doi:10.1038/s41537-024-00462-4)
Supplement: Supplementary file 1 — Supplementary material 1 [file 41537_2024_462_MOESM1_ESM.docx]

Figure 1: Item Characteristic Curve (ICC) for the tests of the Ethiopian Cognitive Assessment battery in Schizophrenia (ECAS)

Figure 2: Item Information Function (IIF) for the tests of the Ethiopian Cognitive Assessment battery in Schizophrenia (ECAS)
